# The Utilization of Plant Facilities on the International Space Station—The Composition, Growth, and Development of Plant Cell Walls under Microgravity Conditions

**DOI:** 10.3390/plants4010044

**Published:** 2015-01-20

**Authors:** Ann-Iren Kittang Jost, Takayuki Hoson, Tor-Henning Iversen

**Affiliations:** 1 Centre for Interdisciplinary Research in Space (CIRiS), NTNU Samfunnsforskning AS, Dragvoll Allé 38, Trondheim NO-7491, Norway; E-Mail: a.i.kittang.jost@ciris.no; 2 Department of Biology, Graduate School of Science, Osaka City University, Sumiyoshi-ku, Osaka 558-8585, Japan; E-Mail: hoson@sci.osaka-cu.ac.jp; 3 Department of Biology, The Norwegian University of Science and Technology (NTNU), Realfagbygget, Trondheim NO-7491, Norway

**Keywords:** International Space Station (ISS), microgravity, *Arabidopsis thaliana*, European Modular Cultivation System (EMCS), BIOLAB, cell wall, gravity resistance, hypergravity, microtubules, protoplasts

## Abstract

In the preparation for missions to Mars, basic knowledge of the mechanisms of growth and development of living plants under microgravity (micro-g) conditions is essential. Focus has centered on the g-effects on rigidity, including mechanisms of signal perception, transduction, and response in gravity resistance. These components of gravity resistance are linked to the evolution and acquisition of responses to various mechanical stresses. An overview is given both on the basic effect of hypergravity as well as of micro-g conditions in the cell wall changes. The review includes plant experiments in the US Space Shuttle and the effect of short space stays (8–14 days) on single cells (plant protoplasts). Regeneration of protoplasts is dependent on cortical microtubules to orient the nascent cellulose microfibrils in the cell wall. The space protoplast experiments demonstrated that the regeneration capacity of protoplasts was retarded. Two critical factors are the basis for longer space experiments: *a*. the effects of gravity on the molecular mechanisms for cell wall development, *b*. the availability of facilities and hardware for performing cell wall experiments in space and return of RNA/DNA back to the Earth. Linked to these aspects is a description of existing hardware functioning on the International Space Station.

## 1. Introduction

The plant cell wall in a growing plant is composed of a variety of components—cellulose, matrix polysaccharides, phenolics, and glycoproteins [1]. The wall, which surrounds the plant cell, is the major source of mechanical strength and form and despite its rigidity it permits and facilitates growth. The network of cell walls in a plant can be compared to both skin and bones in humans and animals. The cell walls surrounding the plasma membrane define the boundaries of the cell itself and represent the place where the plant cell meets a variety of environmental signals. In this way, the wall is the major location of plant responses to such signals [2,3]. Located at the surface and forming the interface between adjacent cells, the wall plays an important role in cell intercellular communication.

This review focuses on gravity as one of the environmental signals that is always present in a constant direction and magnitude on Earth. Throughout evolution, plants have utilized gravity as the most stable and reliable signal for their survival. The immediate effect of gravity enables plants to orient their leaves towards sunlight and to develop a root system to anchor and absorb water and minerals. Plant gravitropism is the most typical response when a plant is turned onto its side and the plant stem bends upwards towards the gravity direction and the root grows the opposite way. This occurs both in light and in darkness but the true gravitropic response requires darkness. It is also well recognized nowadays that the best environment for studying plant gravitropism in order to elucidate the mechanisms behind gravity effects is under microgravity conditions, *i.e.*, in space experiments.

Gravity resistance was introduced by one of the authors of the present article as a new term, and its nature and mechanism were initially studied in ground-based experiments using hypergravity conditions produced by centrifugation [4,5]. More recently, the same authors performed a space experiment on the International Space Station (ISS) to study the effect of microgravity on the gravity resistance phenomena [6]. These experiments were proposed and performed in order to explain that the initial curvature, the true gravitropism, and the following straight growth against gravity are quite different in origin and mechanisms. According to the results obtained, the mechanical resistance to the gravitational force may be a principal graviresponse in plants, independent of gravitropism. In the present review article, the results obtained, which have clarified the outline of the sequence of events in gravity resistance, will be described in more detail. The role of the cell wall in gravity resistance in plants will also be further elucidated.

Since the experiments in space are dependent on the availability of plant cultivation facilities, adequate hardware will be described in the present article. Historically, the review starts with a link back to the early plant experiments in the US Space Shuttle period. Initially, and due to the limited time periods in space (up to 15 days), freshly produced plant cell protoplasts were used to study the effect of short exposures to microgravity conditions and the effect on single cells. Plant protoplasts *i.e.*, cells from which the cell wall has been enzymatically removed, were produced immediately before the launch of the space shuttle, and the initial effects of the cell wall regeneration was followed for the first time under micro-g. Further on in the present article, a summary of these results obtained with cell walls is presented. After the introduction of a functional ISS in 2005, cultivation of intact plants originating from seeds was possible. The different types of facilities and their limitations and advantages for intact plant studies focusing on growth and development of the cell walls are presented.

The end of this review article deals with the molecular aspects of the cell wall development under micro-g conditions. In order to fully understand the cell wall composition, function, and regulation on the Earth, it is expected that results from space experiments will be useful. For the optimal construction of such experiments, sophisticated methods within molecular biology, molecular genetics, and genomics will have to be applied. A key question here, which has not yet been answered, is how to obtain and preserve RNA/DNA samples from the microgravity environment for further analysis on Earth.

## 2. Gravity Resistance

Plants show mechanical resistance to gravitational acceleration. Genetic and physiological evidence indicate that this response, termed gravity resistance, is distinct from gravitropism. Weeping, characterized by hanging branches, is caused by a genetic mutation. Weeping is not a mutant of gravitropism, because branches of most weeping trees show normal gravitropism [7]. However, weeping branches lose the capacity to maintain enough mechanical rigidity to resist gravitational acceleration [8]. On the other hand, stem organs of some aquatic or semi-aquatic plants such as rice coleoptiles elongate rapidly and grow more slender when underwater. This growth response is partly caused by reduction in the gravitational acceleration actually applied to their bodies due to buoyancy, and it is reversible [2,8]. The rigidity of rice coleoptiles when grown under submergence is lower than when grown in the air [2]. However, rice coleoptiles show normal gravitropic curvature under submergence, as in the air [9]. In the gravitropic curvature of stem organs and roots, the initial curvature, which represents true gravitropism, and the following straight growth against gravity are quite different in origin and mechanism. Gravity resistance may sustain the latter process [8]. The cell wall is the major source of mechanical rigidity for plant bodies, and so it may be responsible for gravity resistance. Evidence obtained by the ground-based studies and space experiments described below supports this hypothesis.

## 3. Gravity Resistance and Evolution

During evolution, a link has been created between the responses to various mechanical stresses and the establishment of land plants. When plant ancestors first emerged on land, probably about 450 million years ago in the mid-Ordovician, their bodies were surrounded by soft cell coverings, because it was not necessary for them to develop hard bodies. Plant ancestors were directly exposed to a gravitational acceleration of 1 g on land, and only plants that successfully evolved a tough body with rigid cell walls were able to resist the force and survive on land [10,11]. The development of the mechanisms of gravity resistance was indispensable for the transition of plant ancestors from an aquatic environment to a terrestrial environment and the consequent establishment of land plants. On land, plants also have been exposed to a variety of mechanical stresses, such as wind. An increase in cell wall rigidity is, as for the gravity signal, generally induced in response to mechanical stimuli [12,13,14], the same. In addition, the pathway leading to gravity resistance, described below, may be at least in part common with that of mechanical stresses. Thus, the development of the mechanisms of gravity resistance should be important for the acquisition of responses to mechanical stresses for plants [8].

## 4. Mechanisms Controlling Gravity Resistance

The mechanism of gravity resistance consists of signal perception, transformation and transduction of the perceived signal, and response to the transduced signal, as is seen in other environmental responses as well. The outline of the sequence of events in gravity resistance has already been described [5,15]. The gravity signal may be perceived by mechanoreceptors (mechanosensitive ion channels) on the plasma membrane, and it appears that amyloplast sedimentation in statocytes is not directly involved. The signal should be then transformed and transduced into the cells. Membrane sterols and cortical microtubules (CMTs) may be involved in this process. In the membranes, sterols consist of microdomains called rafts, and CMTs are linked to the rafts via microtubule-associated proteins (MAPs). Because the functions of CMTs, the plasma membrane, and the cell wall are mutually and deeply dependent, as shown by the results of studies using mutants and metabolic inhibitors, the structural continuum or physiological continuity of these cellular components may play a key role in signal transduction. The transduced signal then may induce the expression of diverse genes influencing the structure and function of various cellular components. These steps regulate the synthetic levels of cell wall constituents. At the same time, transformed signals may be transduced through the plasma membrane, and they might modify the activities of proton pumps or other ion channels, which regulates the cell wall environment. The signal transduced through the plasma membrane may also directly influence cellulose synthase activity or exocytosis of Golgi vesicles. In addition, the changes in orientation of CMTs may bring about reorientation of cellulose microfibrils. The modifications to the cell wall environment, in combination with the changes in the levels and structure of the cell wall constituents, determine the cell wall rigidity, leading to gravity resistance.

## 5. Cell Wall Changes under Hypergravity Conditions

Microgravity conditions can be produced by free fall or parabolic flight on Earth. However, the microgravity obtained by these methods is generally too brief to induce obvious changes in plant growth and morphogenesis. Therefore, hypergravity generated from centrifugation has been widely used to analyze plant responses to gravity. Since the pioneering study by Waldron and Brett [16] in pea seedlings, the effects of centrifugal acceleration on growth and development have been analyzed in various plant materials. As a result, hypergravity has been shown to suppress elongation growth but promote the lateral expansion of stem organs and roots [4,5,17]. The first analysis of changes in the mechanical properties of the cell wall under hypergravity conditions was carried out in garden cress seedlings [18]. It has been confirmed in various materials that hypergravity increases cell wall rigidity [4,5].

The structural aspects of the wall under different gravity conditions are the basis for understanding how the gravitational acceleration can influence the mechanical rigidity of the cell wall. The plant cell wall consists of two phases: a cellulosic microfibrillar phase and a matrix phase consisting of polysaccharides, phenolics, and glycoproteins (see e.g., Carpita and Gibeaut [1]). The rigidity of the cell wall is determined by the chemical nature of each wall constituent and the interactions among them, such as cross-link formation. In particular, the levels of wall constituents and the molecular mass of certain matrix polysaccharides are important for the regulation of the rigidity. Hypergravity has been shown to increase cell wall thickness in various organs [4,5,8]. In azuki bean epicotyls, hypergravity increased the level of cellulose microfibrils in the basal supporting regions, and those of matrix polysaccharides in the upper growing regions [19,20]. Cellulose accumulation was also induced by hypergravity in pollen tubes [21]. On the other hand, hypergravity caused the polymerization of certain matrix polysaccharides, the types of which differed between dicotyledonous plants and monocotyledonous *Gramineae* plants. In dicotyledons, hypergravity increased the molecular mass of xyloglucans, whereas hypergravity increased that of 1,3,1,4-β-glucans in *Gramineae* plants. Hypergravity also decreased the xyloglucan-degrading activity in dicotyledons and 1,3,1,4-β-glucanase activity in *Gramineae*. In addition, modifications to xyloglucan metabolism under hypergravity conditions were regulated by prompt and differential changes in the expression of xyloglucan endo-transglucosylase/hydrolase (XTH) genes [22]. In pollen tubes, hypergravity altered intracellular vesicle transport related to the synthesis of matrix polysaccharides [21]. Thus, modification of the metabolic turnover of matrix polysaccharides as well as their accumulation may be involved in making the cell wall mechanically rigid to resist the gravitational acceleration under hypergravity conditions.

Certain differentiated cell walls contain a significant amount of phenolic substances such as lignin and monophenols. The lignin content was increased in response to hypergravity treatment in various stem organs [8]. In wheat seedlings, the levels of monophenols, in particular of diferulic acid, were increased under hypergravity conditions [8]. Also, the activity of wall-bound peroxidase, which is responsible for polymerization of lignin and the coupling reaction that produces diferulic acid, was increased by hypergravity [20,23]. The stimulation of cross-link formation by phenolics may increase cell wall rigidity and thus enhance resistance to the gravitational acceleration.

## 6. Cell Wall Changes under Micro-g Conditions—Space Experiments

Experiments under true microgravity conditions in space are effective to study gravity responses in plants. Opportunities for space experiments have been limited, but the situation was greatly improved by the initiation of scientific operations on the ISS. Growth and development of various organs has been examined in space, but the results are controversial. Such a contradiction may be caused by differences in culture conditions other than gravity, as well as differences in materials, age, and parameters measured [4,5,17]. Careful consideration of these conditions has confirmed growth stimulation under microgravity conditions in space at least in seedlings of Arabidopsis and rice and in inflorescence stems of Arabidopsis [4,5,24,25]. The first analysis of changes in cell wall rigidity under microgravity conditions in space was carried out in RICE experiments on the Space Shuttle STS-95 mission, and it was confirmed that microgravity decreased cell wall rigidity in seedlings of Arabidopsis and rice and inflorescence stems of Arabidopsis [4,5,25].

A comprehensive analysis of the chemical properties of the cell wall constituents was also conducted in the RICE experiments. The levels of cellulose and matrix polysaccharides per unit length were lower in the space-grown rice coleoptiles and Arabidopsis hypocotyls than the controls, indicating that microgravity decreased the cell wall thickness. The space-grown materials also had a lower molecular mass of the matrix polysaccharides, which is mainly due to the decrease in the molecular size of 1,3,1,4-β-glucans in rice and xyloglucans in Arabidopsis. In Arabidopsis hypocotyls, the activity of xyloglucan-degrading enzymes was also increased in space. These changes in cell wall properties are opposite to those induced by hypergravity, as mentioned above. These results support the hypothesis that under microgravity in space the metabolic turnover of cell wall constituents of plant seedlings is activated, which leads to a decrease in cell wall rigidity [4,5,8].

As to phenolics, the changes in content and formation of lignin under microgravity conditions in space were first analyzed in hypocotyls of pine and mung bean [26]. The lignin content in space-grown materials was lower than in those grown at 1 g on Earth. Also, peroxidase activity in space-grown pine hypocotyls was lower than that of ground controls [26]. On the contrary, there were no significant effects of microgravity on lignin content in seedlings or leaves of wheat [27,28]. This discrepancy may be due to the differences in experimental conditions mentioned above.

## 7. Cell Walls and Gravity Resistance—Future Aspects

The results obtained by the ground-based studies and space experiments so far support the hypothesis that plants increase the rigidity of their cell walls via modifications to the metabolism of cell wall constituents to resist the gravitational acceleration. However, the type of plant materials used in space experiments is limited. Changes in cell wall properties need to be examined in further space experiments to confirm the universality of cell wall functions in gravity resistance. On the other hand, CMTs are involved in gravity responses in plants through the regulation of the orientation of cellulose microfibrils, as discussed below for the protoplasts. Under hypergravity conditions, the expression of most α- and β-tubulin genes was upregulated in Arabidopsis hypocotyls, depending on the magnitude of the gravitational force [29,30], and a reorientation of CMTs from transverse to longitudinal directions was induced [31]. To clarify the role of CMTs in gravity resistance, we are conducting Resist Tubule (PI, T. Hoson) and Aniso Tubule (PI, K. Soga) space experiments on the Japanese Experiment Module “Kibo” of the ISS.

The physiological functions of the cell wall are sustained by its intimate crosstalk with the symplast, and the plasma membrane plays an important role in regulating the crosstalk [15]. As mentioned above, mechanoreceptors located on the plasma membrane may be responsible for the perception of the gravity signal, and membrane rafts play a role in transformation and transduction of the perceived signal in gravity resistance. In addition, two other plasma membrane components, cellulose synthase complexes and H^+^-ATPase (proton pump), are likely involved in gravity resistance [5,8,15]. The role of these membrane components and the interaction among them in gravity resistance remain to be clarified in future space experiments.

## 8. Micro-g and Cell Wall Free Plant Cells (Protoplasts)

Historically, the review of the plant space experiments starts with a link back to the US Space Shuttle period with focus on short stays in space and the effect on single cells. Important here is the use of plant protoplasts *i.e.*, cells from which the cell wall has been enzymatically removed. These cells represent a uniform population of true single cells where tissue specific variations are eliminated. The totipotency of the plant cell makes the protoplasts isolated from mature differentiated tissue competent to express genes that regulate mitotic activity. The lack of cell-to-cell interactions in newly isolated protoplasts makes them ideal objects for environmental changes and systematic modification of extracellular conditions. The effect of variations in gravity forces on cell wall regeneration can easily be detected in the initial stages of the protoplast wall reconstitution. In this context, it should be stressed that this is dependent on CMTs, which are believed to orient the nascent cellulose microfibrils in the regenerating cell wall.

The earliest experiments using protoplast cells started on a satellite launched from the Soviet Union in 1989 [32]. Rapeseed protoplasts were used and exposed for micro-g conditions for 14 days on Biokosmos 9 (Kosmos 2044). The major result of the space experiment was a significant decrease in the content of cellulose and hemicellulose. A callus culture was established from the protoplasts, and after retrieval a decreased number of peroxidase isoenzymes and a decreased total peroxidase activity were also detected. The callus cultures established during the two-week-long flight also showed a retarded growth and division compared to the ground control [32,33]. An attempt to get the retrieved tissue cultures to differentiate into mature plants was unsuccessful. Protoplasts exposed to simulated 0 g for 3 days on a 2-D clinostat and protoplasts, which were permitted to regenerate cell walls at ground conditions (1 g), were used as controls for the Biokosmos 9 experiment. A disadvantage of the Biokomos 9 experiment was, however, the lack of a 1 g centrifuge on board the space vehicle. This meant that it was difficult to differentiate between the effect of the weightlessness and the other environmental conditions (e.g., cosmic radiation) under space conditions. Later on, Skagen [34] attempted to clarify the effect of weightlessness compared to the other effects by using rapeseed protoplasts exposed to simulated 0 g.

The Biokosmos 9 experiment was followed by an experiment called “PROTO” which was flown on the Space Shuttle in 1992 with the IML-1 (International Microgravity Laboratory) mission [35,36,37]. Four years later, a new mission (S/MM-03; Shuttle Mission to Mir-03) was launched where a protoplast regeneration experiment was included. Both the IML-1 and the S/MM-03 confirmed some of the results obtained in Biokosmos 9; after 8–11 days of micro-g conditions the regeneration of the rapeseed protoplasts was retarded and mature plants did not develop from the retrieved calli obtained from the flight samples. In the space shuttle experiments, a centrifuge was placed on board the vehicle and functioned as a 1 g control. In addition, a 1 g ground control was used in the comparison to the micro-g results obtained.

The space flight protoplast experiments have shown that the critical step in the regeneration of the protoplast into an intact cell is the immediate reconstitution of the cell wall. In the period after the enzymatic removal of the cell walls from the mother hypocotyl plant tissue, the intention was that the cell-wall-free cells should be exposed to the micro-g conditions in space. For technical reasons, the protoplast samples in the experimental set-up were permitted to be placed in the shuttle cargo as a late access delivery. This meant that the freshly isolated protoplasts were only 17 hours old when they were exposed to the micro-g conditions in space for the first time. One of the astronauts on the shuttle had the special task to microscopically observe that the cells still were at the cell wall free stage when the Space Shuttle Biorack laboratory was opened in space. This made it possible to follow the regeneration of the walls throughout the complete space period from an early stage up until the small calli had developed.

## 9. Protoplasts and Cell Wall Regeneration

Already in the 1980s, one of the authors of the present review article knew that after the Space Shuttle period, whereby plant cells were exposed space conditions for short periods, a new era with longer duration cultivation of plants, and not only cells, would come in due time. Our space biology group had already been selected as one of the plant cell study groups with experiments for the Space Shuttle Challenger in late 1986. Challenger was launched for the first time in 1983 and after nine successful flights a disaster occurred, and the practical consequence for our group was that we were offered the Biokosmos 9 experiment as an alternative.

Our protoplast experiments and the results obtained and experience gained should eventually lead to preparation of an ISS experiment of longer growth duration. As a link to this work, Skagen [34], in her PhD-thesis and in several articles [38,39,40], had focused on CMTs, which were believed to orient the nascent cellulose microfibrils in the regenerating cell wall [41,42,43,44]. At that time, it was also known that the amount of CMTs was anticipated to affect both the cellulose incorporation [45] and the division capacity in cell cultures [46].

**Figure 1:**
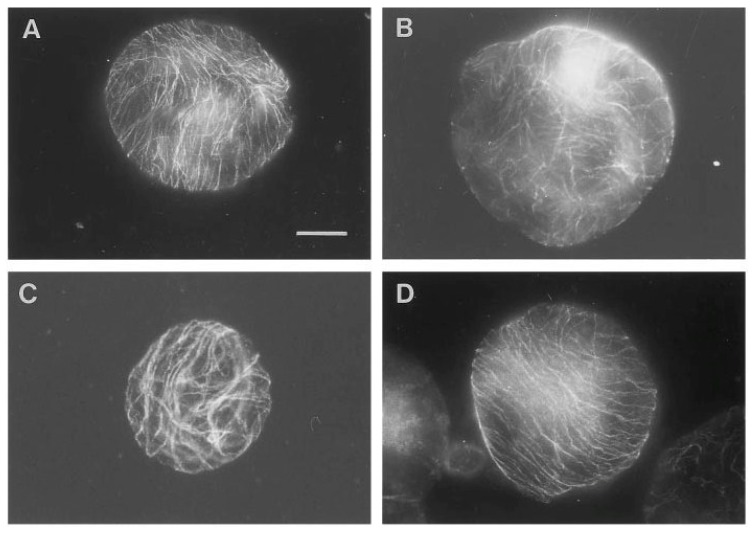
Overview of pattern of corticular microtubules (CMT) in protoplasts exposed to 48 hours of various g-forces. In contrast to most of the protoplasts exposed to 1, 7 or 10 g, which reoriented the CMT in parallel arrays (illustrated by the 1 g- (**A**) and 10 g-protoplast (**D**)), the 0 g-protoplasts showed CMT aligned in a random pattern after 48 h of g-treatment (**B**). Protoplasts with partly parallel arrays were observed in all groups, here illustrated by the 7 g-protoplast (**C**). An increased size among the 0 g-protoplasts compared to the 1 g- and hyper-g-protoplasts was observed (bar: 20 mm)—(original picture by Skagen and Iversen [40]).

The importance of various g-forces and the orientation of CMTs in the protoplast cell wall regeneration have been described by [40]. As demonstrated in Figure 1 [40], the protoplasts exposed to 1, 7 and 10 g reoriented the CMTs in parallel arrays while protoplasts exposed to simulated 0 g conditions showed CMTs aligned in a random pattern after 48 hours. Based on the experience from the protoplast experiment and the various g-effects on the cell wall regeneration, Skagen [34] selected a number of key elements which should therefore be included in an ISS single cell experiment; the effects of various g-forces and naphtaleneacetic acid (NAA) on the CMT organization, density, wall reconstitution (cellulose synthesis and peroxidase activity), and evaluation of the significance of a dense parallel CMT array for cellulose incorporation and cell division.

A well prepared experimental set-up using rapeseed single cells based on our basic protoplast knowledge was submitted to ESA for tests on the ISS. However, in the final selection of our different proposals submitted to ESA for experiments in the European Modular Cultivation System (EMCS) the use of *Arabidopsis thaliana* as a model plant was given priority. Following is a summary of the adequateness of the EMCS for plant growth and development studies in general and the potential for future plant cell wall studies.

## 10. The EMCS and Plant Space Studies 

**Figure 2:**
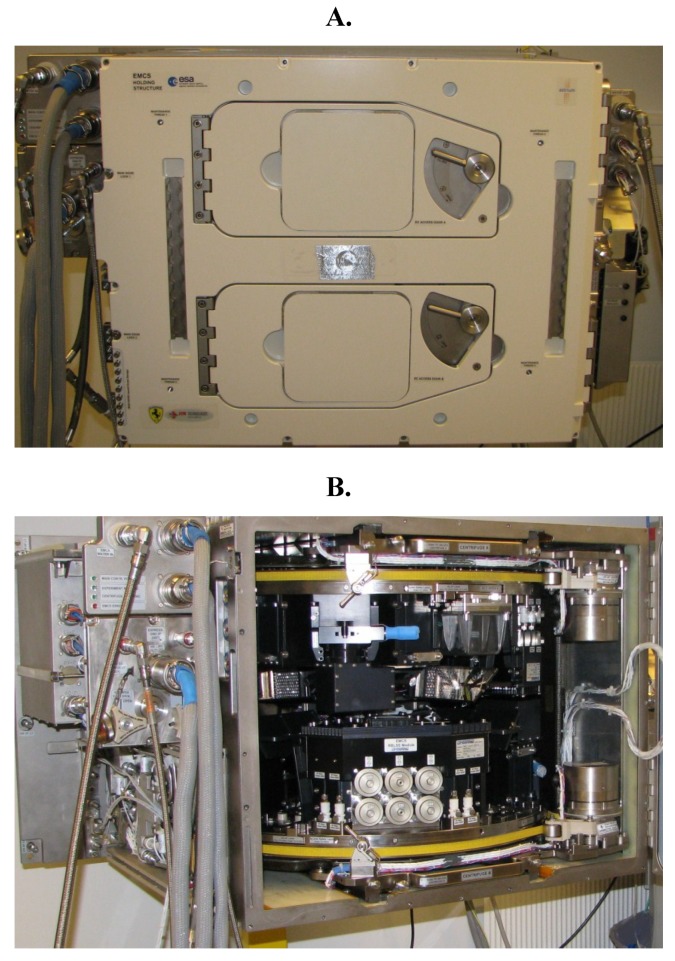
The EMCS Flight Module seen from the front side (**A**) with two doors behind which there are two identical rotors (centrifuges). In (**B**), where the doors are open, a detailed view of the inside of the EMCS is shown. For more detailed descriptions of the EMCS please see [48,49,50,51,52].

The EMCS (Figure 2) and also the parallel space laboratory called BIOLAB were the result of a request to the ESA (the European Space Agency) from plant space scientists, and they were developed by EADS Astrium (Friedrichshafen, Germany) on behalf of ESA. The idea was to design two dedicated facilities to improve and standardize plant growth in the ISS. The EMCS Flight Model (EMCS FM) was brought to the ISS on the Space Shuttle ULF 1.1 in March 2006 after bio-compatibility and functionality tests [47]. The EMCS, which is the facility specially equipped for plant studies, has been equipped with two centrifuges to perform experiments in microgravity with variable *g*-levels up to 2.0 g. Detailed descriptions of the EMCS before being launched have been given by various authors [48,49,50,51,52]. In a more recent review article [53], the utilization of the EMCS on the ISS is presented.

**Figure 3:**
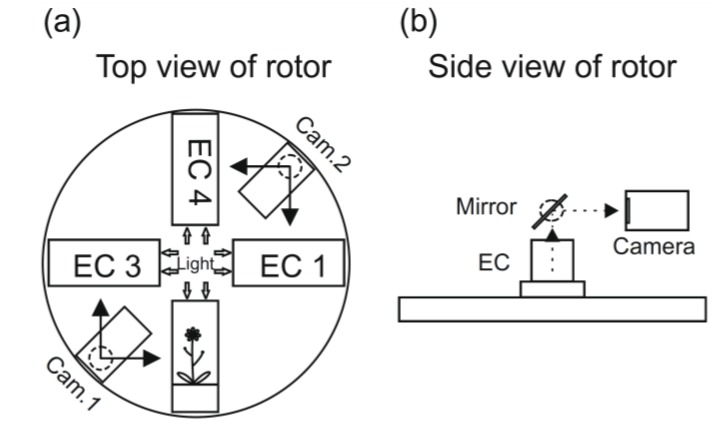
A schematic presentation of the rotor arrangement European Modular Cultivation System (EMCS) (**a**). The ECs are the plant cultivation chambers, and the plants can be photographed using the movable video cameras and with the help of mirrors (**b**) (see [53]).

**Figure 4:**
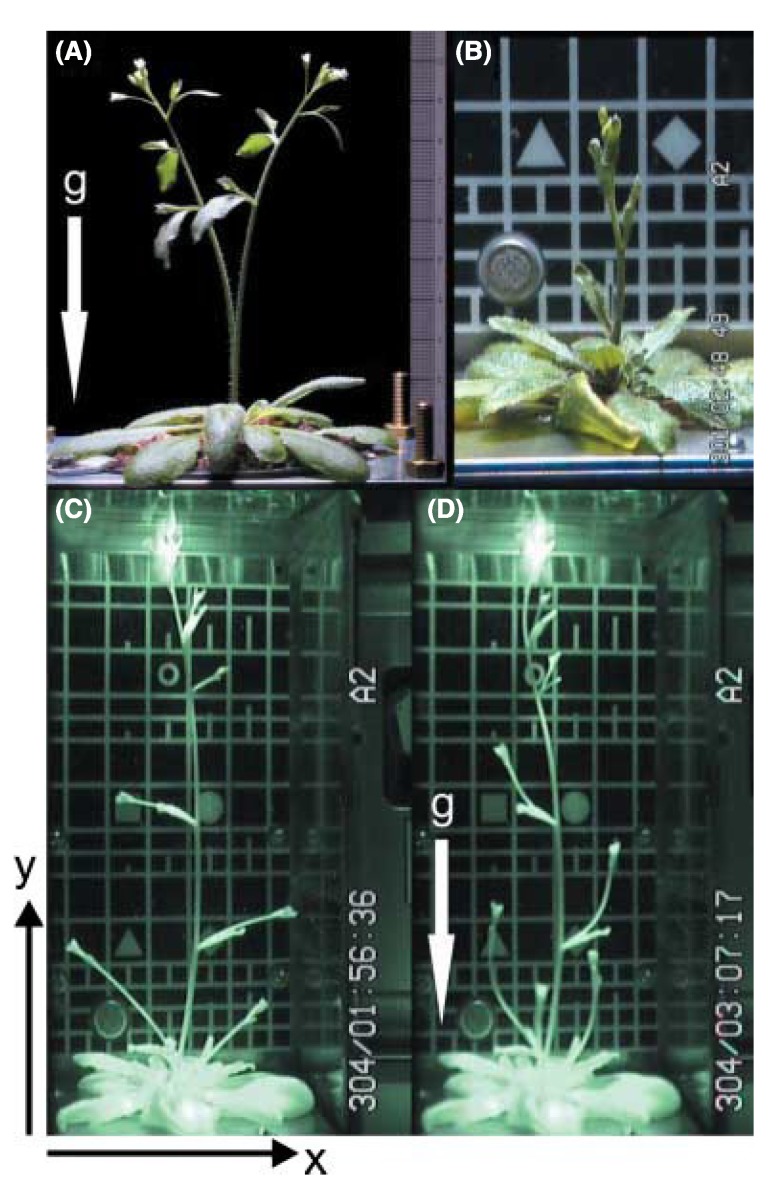
The picture shows an *Arabidopsis thaliana* plant growing in weightlessness on board the ISS in the plant cultivation chamber in the EMCS. The pictures show the different developmental stages and the effect of variable g-forces the plant can be exposed to (micro-g to 0.8 g): (**A**) 1 g on Earth with the force direction as illustrated by the arrow; (**B**) micro-g on the ISS; (**C**) micro-g on the ISS just before the centrifuges are started; (**D**) the same plant as in picture C, 70 min after the centrifuges are started, giving 0.8 g in the middle of the growth chamber with directions as illustrated by the arrow. For further details, see [58].

The experience gained after 8 years of the EMCS in function on the ISS is that cultivation of higher plants can be performed under fully controlled environmental conditions and for long growth periods. So far, ten plant experiments have been performed (Table 1). One of these, the MULTIGEN 1 experiment, lasted for 85 days and *Arabidopsis thaliana* were grown from seeds to new plants producing embryos. In this specific experiment, circumnutations were in focus and, using the video technique (Figure 3) developed for that experiment, detailed representation of their growth and development could be followed over the total growth period as illustrated in Figure 4.

**Table 1:** Experiments performed in the EMCS.

Experiment	Principal investigator	Execution year	Status
TROPI-1	John Z. Kiss	2006-7	[54,55]
GRAVI-1	Dominique Driss-Ecole	2007	[56]
MULTIGEN-1	Tor-Henning Iversen	2007	[57,58]
Cell Wall/Resist Wall	Takayuki Hoson/ Kazuhiko Nishitani	2008	[6]
GENARA-A	Eugenie Carnero-Diaz/F. Javier Medina	2010	Experiment performed
TROPI-2	John Z. Kiss	2010	[59,60]
Plant Signalling	Imara Perera	2011	Experiment performed
Seedling Growth-1	John Z. Kiss	2013	[61]
GRAVI-2	Valérie Legué	2014	Experiment performed
Seedling Growth-2	John Z. Kiss/F. Javier Medina	2014	Experiment performed

## 11. Space Experiments and Cell Wall Behavior

Despite the fact that the ISS has been in function since 2006 and quite a few plant experiments have been performed, the majority of these are already linked to the effect of micro-g on higher plant development. However, principally, samples could have been taken to observe the behavior of the cell wall characteristics, but so far this has not been implemented in any of the ten experiments (Table 1) performed in the EMCS.

The Cell Biology Experiment Facility (CBEF) installed in the Kibo Module also has been used for plant cultivation up to 62 days [62]. As described previously, in the present review article, the growth and cell wall mechanical properties of *Arabidopsis* grown for 33 days were followed in the Space Seed experiment on the Kibo Module on board the ISS [8]—for more detail on the results obtained see Section 6 “Cell Wall Changes under Micro-g Conditions—Space Experiments” in the present review. For the scientific community interested in the effect of various gravity signals on the cell wall behavior, there are possibilities for using both the EMCS and the CBEF on the ISS. However, due to the selection procedure for potential cell wall studies and the long preparation period for ISS experiments, the present authors would advise potential cell wall scientists to contact us in an attempt to implement new relevant experiments in already accepted space experiments using the EMCS (e.g., Plant Development).

## 12. The Effects of Gravity on the Molecular Mechanisms for Cell Wall Development

Global gene expression analysis is a powerful tool to evaluate how plants adapt to microgravity. There have been a few space experiments where the transcriptome of higher plants and cell cultures have been compared with ground controls [60,63,64]. Stutte and co-authors [28] reported that 820 of the analyzed genes were found to be expressed both under microgravity and in the ground control samples, but none of these were found to be above the two-fold cut-off. Etiolated Arabidopsis seedlings and undifferentiated cultured Arabidopsis cells have been exposed to microgravity during the BRIC-16 flight on STS-131. The post-flight analyses revealed transcription profiles that were quite similar for the cells and tissues. The genes regulated a five-fold or less in microgravity, were mainly associated with pathogen response and wounding. The response was moderate in the seedling, but more dramatic for the cell culture. Genes associated with cell wall metabolism and cell elongation were among the genes encoding transcription factors that were down regulated five-fold. As part of the TROPI-2 experiment [59] in EMCS, Arabidopsis seedlings were used to perform transcriptome analyses [60]. The experiment was primarily designed to study tropisms of plants using image analysis, and the preservation to fix the seedlings for RNA extraction on the rotor immediately following the experiment was not feasible due to hardware limitations. Still, by comparing gene expression profiles from a range of gravity treatments, they were able to identify genes involved in e.g., cell-wall development. Among the genes identified when comparing spaceflight and ground were e.g., expansin-related genes (ATEXPA4, ATEXPA7), three extensin-related genes (At4g08380, At5g06630 and LEUCIN-RICH REPEAT/EXTENSIN 1-LRX1) and pectinases and chitinases. When comparing the transcriptome of all the g-treated spaceflight samples with the microgravity samples, only 27 genes were at least two-fold differently regulated and among them was a cell wall kinase (WAK3, At1g48100).

The gene expression studies in space experiments are challenging because the time period from the end of an experiment (*i.e.*, after the stop of the 1× g rotor) until the point in time when the samples are preserved is critical. Studies have shown that genes are induced by gravity and mechanical stimulation less than 2 min in the Arabidopsis root apex [65]. The time period from the end of an experiment (*i.e.*, after the stop of the 1 × g rotor) until the samples are preserved is critical for gene expression studies. The BPS growth system on ISS allows for a sample collection and preservation within 2 min [28]. For samples in the EMCS, the preservation will vary depending of what kind of unique experiment equipment is used. When using equipment like MULTIGEN-1 and TROPI (see Table 1), approximately 1–1.5 h must be anticipated from the point in time when the rotor stops until the last sample is preserved. This depends on how quickly the samples can be removed from the EMCS Experimental Container and be inserted in e.g., MELFI (Minus Eighty Laboratory Freezer for ISS; [66]). When using GRAVI-2 equipment (optimized for experiments on etiolated lentil seedlings), the preservation can take place automatically when the samples are still under a controlled environment and a selected gravity level. A modified version of the GRAVI-2 hardware are being developed to fly the Plant Development experiment that will allow optimal growth of *Arabidopsis thaliana* seedlings under light conditions with preservation in e.g., RNAlater under a controlled environment [53]. When testing the preservation protocol for the MULTIGEN-2 experiment (finally included as part of the Plant Development experiment), the RNA quality and quantity was satisfactory for microarray analysis [66]. However, among the genes induced in the RNAlater preserved samples, three salt inducible transcription factors (ZAT10, SZF1, and SZF2) were identified, suggesting that the high salt concentration in RNAlater causes salt stress before the transcription has stopped. The results do indicate that not all the biological processes are stopped instantly by the RNAlater, and further optimization of the preservation protocol will be performed.

The Random Positioning Machines (RPMs) and clinostats allow studies of simulated microgravity effects on plants on the ground. A RPM consists of two planar frames that can be rotated independently with random speeds and directions [67,68,69]. In several cases, it has been demonstrated that higher plants grown on RPMs perform a similar growth and cellular response as under true microgravity conditions for the parameters studied [24,68,70]. When exposing *Arabidopsis thaliana* seedlings on a RPM under light conditions for 16 h the results demonstrated a moderate to low regulation of 55 genes (<0.2% of the analyzed genes; [71]). Most of the differentially expressed genes could be linked to either a stress or light response. The AtEXP8 was among the significantly repressed genes, and this gene encodes an expansin with a known cell-wall loosening function in plants [72] linked to auxin and both photo- and gravitropic growth responses [73]. Gene expression analyses on a DNA chip have been carried out on 6 day old plants using a 3-D clinostat in two experiments, the first lasting for 1 h and the second for 6 days [74]. Both the 1 h and the 6-day-long exposures showed a differential expression of a relatively large number of genes (400 and 500 genes, respectively). Among the induced genes were Ext (At3g244890) that has been linked to cell wall modifications and plant tip growth [75]. This gene was shown to be induced after a 1 h exposure to simulated microgravity, but not after a 24, 96 or 144 h exposure. This indicated an early response for redistribution/reformation of the composition in the cell wall structures [74]. In the same study, the XTR6 (At4g25810) gene was found to be significantly induced, especially after 24 h. The XTR6 correlates with plant cell expansion/elongation by decorating cellulose micro fibrils and forming cross-links between them [76].

## 13. Conclusions

The results obtained from the ground-based studies and space experiments have supported the hypothesis that plants increase the rigidity of their cell walls via modifications to the metabolism of cell wall constituents in gravity resistance. Because the type of plant materials used in space experiments is limited, the universality of this hypothesis needs to be confirmed in further space experiments. In addition, the roles of the symplast and the plasma membrane sustaining the functions of the cell wall remain to be clarified.

The earliest space experiments using rapeseed protoplasts exposed for micro-g conditions for 14 days on Biokosmos 9 demonstrated a significant decrease in the content of cellulose and hemicellulose. From the protoplasts retrieved after flight, the callus cultures established showed a retarded growth and division compared to the ground control, and the tissue cultures were not able to differentiate into mature plants. Both the IML-1 and the S/MM-03 experiments also using protoplasts confirmed some of the results obtained in Biokosmos 9; after 8–11 days of micro-g conditions the regeneration of the rapeseed protoplasts was retarded and mature plants did not develop from the retrieved calli obtained from the flight samples. The space flight protoplast experiments have shown that the critical step in the regeneration of the protoplast into an intact cell is the immediate reconstitution of the cell wall. CMTs are believed to orient the nascent cellulose microfibrils in the regenerating cell, and apparently the amount of the CMTs is anticipated to affect both the cellulose incorporation and the division capacity in cell cultures.

The results from the performed space experiments, including the few focusing on cell walls, are difficult to interpret and often inconclusive due to the use of various flight hardware, growth conditions, preservations, the small number of experiments, and few replicates. Future space experiments should therefore have standardized conditions e.g., for plant growth [77,78]. Whenever possible, the preservation for gene expression analyses should take place under environmentally controlled conditions. For the “omic data” (e.g., genomics and proteomics), a general database should be developed [77] giving the science community access to these limited and exclusive space experiment datasets.

Cell wall molecular mechanisms that are well established on Earth (e.g., cell wall loosening and selective cell wall rearrangement mechanism) are important candidates for space experiments.
